# Parallel testing of several genes (panel-testing) in patients with ectodermal dysplasia using next-generation sequencing

**DOI:** 10.1186/1746-160X-8-S1-O1

**Published:** 2012-05-25

**Authors:** HD Gabriel, E Decker, M Gencik

**Affiliations:** 1 diagenos: Humangenetische Praxis, Osnabrück, Germany

## 

Ectodermal dysplasia (ED) is a group of syndromes characterized by abnormalities of structures of ectodermal origin. Up to now more than 150 different syndromes are known. Affected ectodermal structures are mainly hair, teeth, nails and sweat glands. ED can be classified by mode of inheritance (autosomal dominant, autosomal recessive and X-linked), by additional clinical symptoms (e.g. facial abnormalities) or by the structures involved. Hypohidrotic or “classical” ED (HED) is caused by mutations in the genes EDA, EDAR and EDARADD. Mutations in the X-linked EDA gene underlie most cases. EDAR and EDARADD are known to be associated with both autosomal dominant and autosomal recessive forms of HED. As many ED forms show overlapping clinical phenotypes, often many genes have to be analyzed to identify the causative mutation in a patient, therefore Sanger sequencing frequently takes several months and is laborious and cost-intensive. We have designed an accurate and fast molecular test for the 6 most common ED genes using next-generation sequencing. We developed a novel PCR-based protocol on a Roche 454 Junior platform for mutation screening of the genes EDA, EDAR, EDARADD, TP63, GJB6 and WNT10A. The verification and validation process was finished in October 2011, and the “ED panel” is now the routine diagnostic test for ED in our institute. During the last months several mutations in different genes could be identified with this method. A definitive diagnosis could be made rapidly in a number of so far „unsolved cases”. In summary, the next generation sequencing approach has the potential to increase the diagnostic sensitivity by expanding the number of genes that can be screened in parallel while also reducing expenditure of time and costs.

